# Supplementary material for: HOXB9 Overexpression Confers Chemoresistance to Ovarian Cancer Cells by Inducing ERCC-1, MRP-2, and XIAP
Source: Int J Mol Sci. 2023 Jan 8;24(2):1249. doi: 10.3390/ijms24021249 (PMC9865712; doi:10.3390/ijms24021249)

Supplementary Materials

Supplementary table S1. Catalogue numbers and dilutions of the antibodies used for western blot and immunohistochemistry

Company	Cat#	Antibody	Dillution Factor
Thermo Fisher Scientific	PA5-40576	HOXB9	1/1000
abCAM	ab129267	ERCC-1	1/1000
abCAM	ab187644	MRP-2	1/1000
abCAM	ab182858	BCL-2	1/2000
abCAM	ab182733	BAX	1/2000
Cell Signaling Technology	87809	MMP-2	1/500
Cell Signaling Technology	13667	MMP-9	1/500
Cell Signaling Technology	14472	E-Cadherin	1/1000
Cell Signaling Technology	2808	Survivin	1/500
Cell Signaling Technology	14220	Caspase-3	1/1000
Cell Signaling Technology	9661	Cleaved Caspase-3	1/1000
Cell Signaling Technology	20750	Cleaved Caspase-9	1/1000
Cell Signaling Technology	9532	PARP	1/1000
Cell Signaling Technology	5625	Cleaved PARP	1/1000
Cell Signaling Technology	14334	XIAP	1/500
Cell Signaling Technology	2144	α-tubilin	1/5000

Bcl-2, B-cell lymphoma 2; Bax, BCL2 associated X protein; E-Cadherin, epithelial cadherin; ERCC-1, excision repair cross-complementing rodent repair deficiency, complementation group 1; HOXB9, homeobox B9; MMP-2, matrix metalloproteinase-2; MMP-9, matrix metalloproteinase-9; MRP-2, multidrug resistance protein 2; PARP, Poly (ADP-ribose) polymerase; XIAP, X-linked inhibitor of apoptosis protein

Supplementary Table S2. Primers used for reverse transcription polymerase chain reaction

Genes	Primers	Sequences (5'--->3')	Product size (bp)	Annealing Temp. (℃)	#PCR Cycles
HOXB9	Forward	GGTGGGCTAGAAAGTACAAGAAAA	298	62	28
	Reverse	GATACGACCGAAGAGAGTTGATTT			
E-Cadherin	Forward	AATGTTTATTTAGCTCTGAAGCAAGAG	369	55	30
	Reverse	AATTTTGAATATTCTCGGTTTTCTGT			
N-Cadherin	Forward	GGAGACATTGGGGACTTCATTA	221	61	30
	Reverse	CCACCATACATGTCAGCAAGTT			
Vimentin	Forward	TGACATTGAGATTGCCACCTAC	206	60	32
	Reverse	GAGATGCTGAGAAGTTTCGTTG			
VEGF	Forward	TAAAATTCATGTTTCCAATCTCTCTCT	196	56	30
	Reverse	ATACACACAAATACAAGTTGCCAAATA			
IGF	Forward	CAGGGGCTTTTCTTTCAACAAG	230	60	28
	Reverse	TCGTGTTCTTGTTGGTAGATGG			
Nanog	Forward	GTGATGAAGATGTATTCGTATTGTTTG	251	58	33
	Reverse	AGGAAAAATAGTTGTTTCATTCATTTG			
Oct-4	Forward	CTCACTTCACTGCACTGTACTCCT	205	62	33
	Reverse	TAGTGAATGAAGAACTTAATCCCAAAA			
Sox-2	Forward	AACGTGAAAAGAAGAAAATTATTCAAA	208	61	30
	Reverse	AGCTATTATAAATTACCAACGGTGTCA			
Bax	Forward	CCCGAGAGGTCTTTTTCCGAG	154	56	25
	Reverse	CCAGCCCATGATGGTTCTGAT			
Bcl-2	Forward	ATACTGAGTAAATCCATGCACCTAAAC	273	62	28
	Reverse	AACAAAAGCTTTCAATACAAAAACACT			
18s rRNA	Forward	GAGCGAAAGCATTTGCCAAG	100	60	25
	Reverse	GGCATCGTTTATGGTCGGAA			

Bcl-2, B-cell lymphoma 2; Bax, BCL2 associated X protein; E-Cadherin, epithelial cadherin; ERCC-1, excision repair cross-complementing rodent repair deficiency, complementation group 1; HOXB9, homeobox B9; IGF, insulin-like growth factor; MMP-2, matrix metalloproteinase-2; MMP-9, matrix metalloproteinase-9; MRP-2, multidrug resistance protein 2; Oct-4, octamer-binding transcription factor 4; PARP, Poly (ADP-ribose) polymerase; Sox-2, sex determining region Y box-2; VEGF, Vascular endothelial growth factor; XIAP, X-linked inhibitor of apoptosis protein

Supplementary figure S1. Quantification for the protein expression levels of the figure 6E

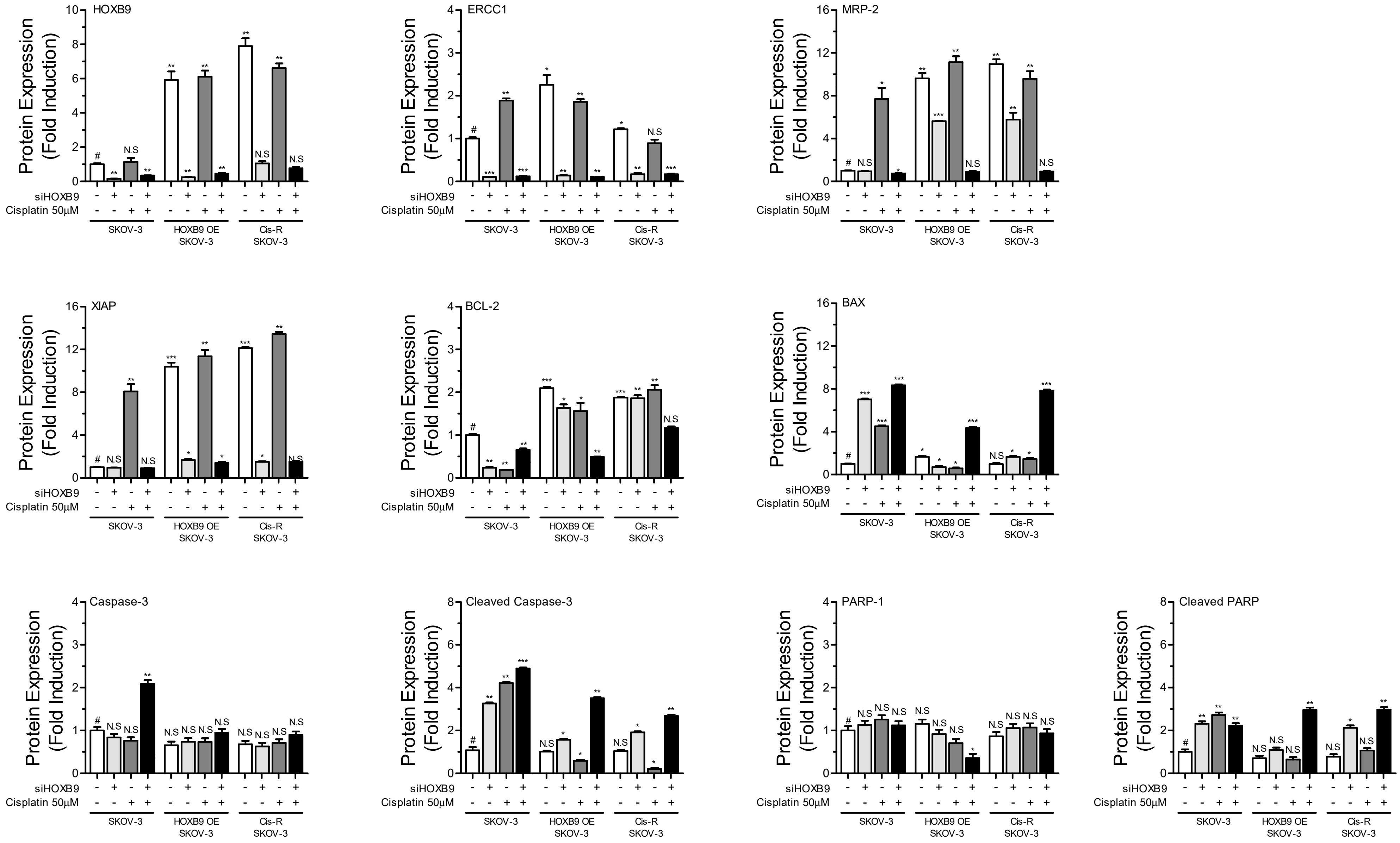

Supplement: Supplementary file 1 [file ijms-24-01249-s001.zip › ijms-2131022-supplementary.pdf]
